# A Case of Suspected Homeopathy-Induced Stevens–Johnson Syndrome in a Pediatric Patient From Bangladesh

**DOI:** 10.7759/cureus.83325

**Published:** 2025-05-01

**Authors:** Nahian Rabby, Soumyadipto B Arko, Utsow Saha, Pooja Roy, Silvia Monjur, Tania Uddin, Arpita Kundu

**Affiliations:** 1 Internal Medicine, Enam Medical College, Savar, BGD; 2 Internal Medicine, Dhaka Medical College Hospital, Dhaka, BGD; 3 Internal Medicine, Icahn School of Medicine at Mount Sinai, Queens Hospital Center, New York, USA; 4 Internal Medicine, Harlem Hospital Center, New York, USA; 5 Internal Medicine, Dhaka National Medical College, Dhaka, BGD; 6 Internal Medicine, Bangladesh Medical College, Dhaka, BGD; 7 Biological Sciences, Stony Brook University, Stony Brook, USA

**Keywords:** bangladesh, dermatology, homeopathy, rare case report, stevens–johnson syndrome

## Abstract

Stevens-Johnson syndrome (SJS) is a rare, life-threatening mucocutaneous reaction predominantly triggered by medications or infections. This case report highlights the first documented instance of SJS induced by homeopathic medicine in Bangladesh. A previously healthy one-year-old individual developed widespread erythematous rash, severe mucosal involvement, and systemic symptoms following the intake of a homeopathic preparation prescribed for a minor ailment. Comprehensive clinical evaluation, detailed history, and exclusion of alternative causes confirmed the diagnosis of homeopathy-induced SJS. The patient was managed with immediate cessation of the suspected medication, intensive supportive care, and corticosteroid therapy, leading to gradual recovery. This case underscores the potential risks of unregulated alternative medications and the need for heightened awareness among clinicians regarding adverse drug reactions from unconventional therapies. It also emphasizes the importance of educating the public on the safe use of alternative treatments. This unprecedented case in Bangladesh serves as a cautionary tale for healthcare practitioners and policymakers, highlighting the need to address gaps in regulating and monitoring homeopathic practices.

## Introduction

Stevens-Johnson Syndrome (SJS) is a rare, immune-mediated condition that begins with prodromal symptoms, such as fever, malaise, and a sore throat, and progresses to widespread mucocutaneous involvement. The hallmark of SJS is the rapid onset of erythematous macules, often accompanied by target lesions, which evolve into blistering and epidermal necrosis, affecting both the skin and mucous membranes. The most commonly involved mucous membranes include the oral cavity, eyes, and genitals. The condition can also lead to significant complications, including ocular sequelae, sepsis, and multi-organ failure, making it a potentially fatal condition if not promptly treated.

SJS is predominantly drug-induced, with medications such as anticonvulsants, sulfonamides, and allopurinol being the most common triggers. However, infections and, more rarely, vaccines can also precipitate the syndrome. Immunologically, SJS is classified as a type IV hypersensitivity reaction, in which cytotoxic T-cells cause widespread apoptosis of keratinocytes, leading to epidermal detachment. The condition occurs most frequently in young adults and older children, with a slight predilection for males. Its incidence is estimated to be one to six cases per million people annually, though it is likely underreported, especially in rural or resource-limited areas [1].

Toxic epidermal necrolysis (TEN), a related condition, is marked by fever, prodromal symptoms, and widespread mucocutaneous involvement with full-thickness epidermal necrolysis. When epidermal detachment involves less than 10% of the body surface area, it is classified as SJS. Detachment exceeding 30% is termed TEN, while involvement between 10% and 30% is referred to as SJS-TEN overlap [2,3].

Treatment primarily involves supportive care, including fluid management, wound care, and control of pain and secondary infections. In severe cases, systemic corticosteroids, intravenous immunoglobulin (IVIG), and biologics may be used, although their efficacy remains controversial. Early diagnosis and management in a specialized burn unit or intensive care setting are critical to improving outcomes.

Reports and data on SJS in Bangladesh are limited, primarily due to inadequate healthcare access in rural areas, which restricts clinicians' ability to diagnose and document such cases. In this report, we explore a pediatric case of SJS triggered by the administration of sulphuricum acidum, a homeopathic remedy. Although homeopathic preparations are typically considered safe due to their extreme dilution, rare adverse reactions can occur, particularly in sensitive individuals. This case highlights the importance of being aware of potential triggers, including alternative and complementary medicines, in the clinical management of SJS.

## Case presentation

**Figure 1:**
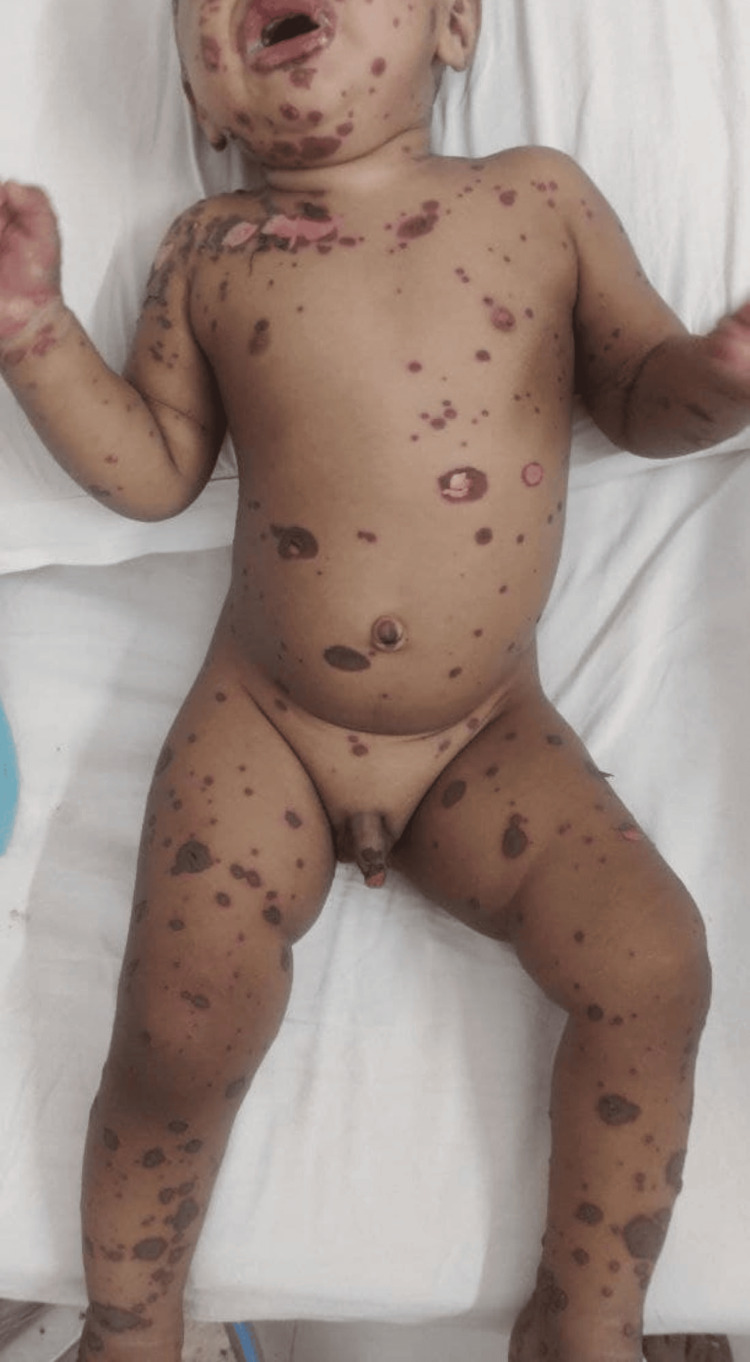
Bullous lesion all over the body

A one-year-old male child, second issue of non-consanguineous parents, presented in the pediatrics department with the chief complaints of multiple bullous lesions all over his body for four days and high-grade, intermittent fever without chills and rigor for the past two days. His father stated that he initially developed an oral ulcer for which he took a homeopathic drug containing sulphuricum acidum. Then, rashes developed on his face, which progressed to multiple bullous lesions all over the body with high-grade fever. Rash was associated with mucosal involvement (eye, oral cavity, urethral orifice and anal orifice). The rashes were dewdrop in appearance, whitish on the inner side and reddish on the border (Figure 1).

On general examination during admission, he was irritable and febrile. Oral ulceration, eye lesion, and multiple ulcerated, desquamated bullous lesions all over the body were noted.

**Figure 2:**
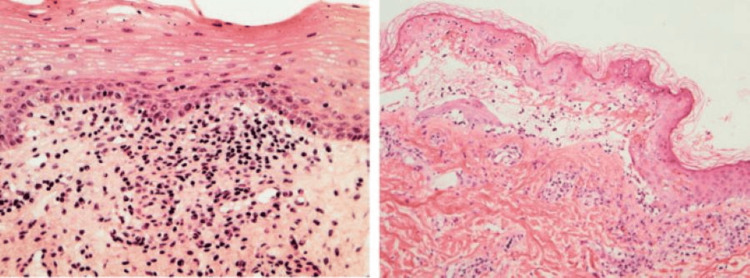
Histopathology of skin showing full thickness epidermal necrosis and separation of the dermis and epidermis

The temperature was 100°F, the respiratory rate was 44 breaths per minute, and the heart rate was 110 beats per minute. The lungs were clear. The abdomen was soft, non-tender, and non-distended. Other systemic examinations reveal no abnormalities. The lab values show an increase in ESR (51 mm in the first hour), total WBC count (13,750/μL), and lymphocyte count (68%), as well as a decrease in neutrophil count (22%). A skin biopsy was obtained (Figure 2), which confirmed the diagnosis of homeopathy-induced SJS. Treated with oral prednisolone, systemic antibiotic intravenous ceftriaxone, famotidine, moxifloxacin eye drops, a sterile eye drop containing polyethylene glycol and propylene glycol, and regular povidone iodine bath. Following the treatment, the response was satisfactory.

## Discussion

SJS, also referred to as "erythema multiforme (EM) major," is a rare but severe form of EM characterized by significant blistering that affects the mucous membranes of the mouth, eyes, and genitalia, often accompanied by systemic symptoms and severe constitutional manifestations [1]. Involvement of the oro-genital region is frequently associated with ulcerations and erosions. In contrast, eye involvement can lead to conjunctivitis and corneal limbal cell deficiency, which may result in visual impairment. Notably, the presence of mucosal lesions is a consistent feature. Skin lesions are widespread, multiple, erythematous, papular, annular, and targetoid (resembling bull’s eyes). The hallmark "target" or "iris" lesion features central pallor or dusky purpura, surrounded by edema and peripheral erythema [2].

Although SJS can be mistaken for conditions such as TEN, EM, or SJS-TEN overlap, the documented case of a child with similar features is consistent with SJS, as less than 10% of the total body surface area was affected, and the clinical presentation aligns with typical SJS characteristics [3]. Notably, SJS can be distinguished from conditions like TEN and EM, as the extent of epidermal detachment in SJS is usually less than 10% of the total body surface area. This case aligns with the typical presentation of SJS, confirming the diagnosis despite some overlapping features with other conditions [2,3].

The pathogenesis of SJS-TEN has been linked to genetic factors, including HLA and non-HLA genes, drug-specific T-cell-mediated cytotoxicity, T-cell receptor (TCR) restriction, and cytotoxic pathways. SJS-TEN is often triggered by medications such as sulfonamides, nitrofurantoin, antiepileptics, and certain nonsteroidal anti-inflammatory drugs [4]. Additionally, toxic skin reactions to sulfur-containing drugs have been well-documented in HIV-infected patients, raising the suspicion that this case may be linked to "sulphuricum acidum." Nonetheless, many factors contributing to epidermal necrolysis, especially those induced by viral infections or autoimmune mechanisms, remain unidentified [5]. The role of homeopathic treatments, including sulphuricum acidum, is less commonly recognized in the literature, though it warrants attention given the temporal association in this case. Despite its extremely dilute concentration, homeopathic preparations can sometimes lead to unexpected immune reactions, underscoring the importance of considering all forms of medication, including alternative treatments, in patients with SJS-TEN.

Management of SJS begins with discontinuing the causative medication. Supportive care includes analgesics, antipyretics, antibiotics, and short-term parenteral fluids for patients with dysphagia. Topical glucocorticoids may help alleviate symptoms, although the efficacy of systemic steroids is debated. Comprehensive supportive measures, including ophthalmologic care and local management of oral lesions, are crucial. In severe cases, IVIG may be considered [6]. Careful monitoring by an ophthalmologist is crucial to prevent long-term ocular complications. Local management of oral lesions is also necessary for comfort and to prevent secondary infections.

This case report emphasizes the need for clinicians to maintain a high level of suspicion for SJS in patients presenting with mucosal lesions and bullous skin eruptions, regardless of the drug or treatment history. The potential for homeopathic medicines to trigger such reactions, though underreported, should not be overlooked. Enhanced awareness and documentation of such responses, particularly to alternative treatments like sulphuricum acidum, could help guide future clinical practice and research on this rare but serious condition [6].

## Conclusions

SJS-TEN, though relatively rare, presents a significant threat to patient health due to its high morbidity and mortality rates, which underscores the urgent need for prompt recognition and intervention. Early identification of the causative drug and its prompt discontinuation are crucial, as they have been shown to dramatically reduce the incidence and severity of these conditions. This highlights the critical importance of obtaining a thorough and accurate drug history, including over-the-counter and alternative medicines, for every patient presenting with suspected SJS-TEN. Although reports of homeopathy-induced SJS are scarce and not widely recognized in clinical practice, clinicians must remain vigilant to the possibility of such adverse reactions, particularly in cases where patients have used homeopathic treatments. This case underscores the need for heightened awareness and meticulous documentation of adverse reactions associated with homeopathic remedies such as sulphuricum acidum, which, despite being highly diluted, can still elicit harmful immune responses. Furthermore, this report calls for expanded research into less-explored etiologies, including those associated with alternative and complementary medicines. Such research could provide a more comprehensive understanding of SJS-TEN pathogenesis and help develop targeted preventive strategies. To address these challenges, there is a pressing need to improve clinician education on recognizing and managing SJS-TEN and to enhance systems for reporting adverse drug reactions. These efforts would not only help reduce the burden of these life-threatening conditions but also foster future research to better identify their triggers, improve clinical outcomes, and ultimately prevent or mitigate the devastating effects of SJS-TEN.
